# SARS CoV-2 infections in animals, two years into the pandemic

**DOI:** 10.1007/s00705-022-05609-1

**Published:** 2022-10-07

**Authors:** Sara Frazzini, Massimo Amadori, Lauretta Turin, Federica Riva

**Affiliations:** 1 Department of Veterinary Medicine (DIMEVET), University of Milan, Milan, Italy; 2 Italian Network of Veterinary Immunology, Brescia, Italy

## Abstract

In December 2019, several cases of pneumonia caused by a novel coronavirus, later identified as SARS-CoV-2, were detected in the Chinese city of Wuhan. Due to its rapid worldwide spread, on 11 March 2020 the World Health Organization declared a pandemic state. Since this new virus is genetically similar to the coronaviruses of bats, SARS-CoV-2 was hypothesized to have a zoonotic origin. Within a year of the appearance of SARS-CoV-2, several cases of infection were also reported in animals, suggesting human-to-animal and animal-to-animal transmission among mammals. Natural infection has been found in companion animals as well as captive animals such as lions, tigers, and gorillas. Among farm animals, so far, minks have been found to be susceptible to SARS-CoV-2 infection, whereas not all the relevant studies agree on the susceptibility of pigs. Experimental infections have documented the susceptibility to SARS-CoV-2 of further animal species, including mice, hamsters, cats, dogs, ferrets, raccoon dogs, cattle, and non-human primates. Experimental infections have proven crucial for clarifying the role of animals in transmission and developing models for viral pathogenesis and immunotherapy. On the whole, this review aims to update and critically revise the current information on natural and experimental SARS-CoV-2 infections in animals.

## Introduction

Infectious diseases affect humans as well as animals, whether domestic or wild. In recent decades, factors such as globalization and urbanization have allowed the spread of new pathogens, with a consequent increase in the number of emerging zoonotic infectious diseases originating from wildlife. This probably applies to the latest episode that occurred in December 2019, when several cases of atypical pneumonia were reported in China, in Hubei’s capital city, Wuhan. In January 2020, a novel betacoronavirus was identified as the causative agent. Based on genetic analysis, the International Committee for Taxonomy of Viruses named it SARS-CoV-2, while the disease was named COVID-19 by the World Health Organization (WHO) [1]. Despite the Chinese authorities' efforts to curb the circulation of the virus, it spread throughout the world, and on 11 March 2020 WHO declared a pandemic state. By January 2022, SARS-CoV-2 had caused the death of over 5 million people out of 364,191,494 confirmed cases world wide since the beginning of the pandemic [2]. The primary source of SARS-CoV-2 was initially thought to be linked to the Huanan Wuhan seafood market, where live wild animals such as birds, snakes, and marmots were on sale [3]. Subsequently, this theory was questioned, and research is currently widening the studies to investigate the origin of the virus.

Studies have shown that the genome of SARS-CoV-2 is similar to that of SARS-CoV-1 (79.6% sequence identity), the virus that caused the 2002-2003 SARS epidemic. Since SARS-CoV-2 shares 96.2% sequence identity with the bat coronavirus RaTG13 (BatCoV RaTG13), SARS-CoV-2 was hypothesized to be zoonotically derived from bats, although this hypothesis has not been confirmed yet [4]. Despite the genomic similarity, the receptor binding domain (RBD) of BatCoV RaTG13 is quite different from that of SARS-CoV-2. It is therefore unlikely that the pandemic virus jumped directly from bats to humans. Probably, due to selective pressure, the SARS-CoV-2 RBD evolved in an intermediate animal, such a fur animal (raccoon dog and/or mink) before its passage to humans. In this regard, one of the animals suggested as a potential intermediate host is the pangolin, because of the high similarity between the human SARS-CoV-2 RBD and the orthologous Malayan pangolin receptor [5]. In order to clarify the possible role of animals in the transmission of SARS-CoV-2, we discuss confirmed cases of COVID-19 in companion, livestock, laboratory, and wild animals. The possible role of COVID-19 vaccines in susceptible animals and the possible contribution of animals to immunoprophylaxis of COVID-19 are also addressed.

## Summary of the pathogenesis of COVID-19 in humans

SARS-CoV-2, a positive-sense, single-stranded RNA virus belonging to the genus *Betacoronavirus*, is responsible for COVID-19 [6]. Transmission mainly occurs through exposure of the respiratory tract to the virus, either directly, through contact of contaminated hands with eyes, and the subsequent passage of the virus through the nasolacrimal duct, or nose, or indirectly, through inhalation of contaminated droplets released by an infected person coughing or sneezing [7].

The virus reaches the lungs through the respiratory tract after passing the mucous membranes of the upper respiratory tract, where it begins to replicate (primary amplification); then, the virus often reaches the lungs, where it further replicates (secondary amplification) and enters the bloodstream, which enables the virus to reach other target organs [8]. Since the internalization of the virus occurs through the binding to the cellular receptor known as angiotensin-converting enzyme 2 (ACE2), all of the target organs express this receptor: lungs, heart, blood vessels, kidney, and gastrointestinal tract [7, 9]. ACE2 is expressed by multiple human cell types, such as type II alveolar cells (AT2), oral, esophageal, and ileal epithelial cells, myocardial cells, proximal tubule cells of the kidneys, and urothelial cells of the bladder [4]. The viral protein involved in cell entry is a glycoprotein spike trimer (S protein, SP), which undergoes proteolytic cleavage, which is necessary to extrude the RBD-containing S1 region after binding to ACE2 [8]. In addition to that, Wang *et al.* have identified an alternative entry route, through the binding of SARS-CoV-2 SP to CD147 [10]. CD147, also known as basigin or EMMPRIN, is a membrane glycoprotein of the immunoglobulin superfamily that is involved in tumor development, *Plasmodium* invasion, and bacterial and viral infections [11]. It is expressed by epithelial cells, endothelial cells, and leukocytes [11].

In approximately 80% of cases, the disease is mild and confined to the upper respiratory tract [12]. The remaining 20% of patients experience virus invasion of the lungs, which often gives rise to severe interstitial inflammation caused by vascular injury [13]. The virus infects alveolar cells, compromising the gas exchange and the renin-angiotensin system. Together with direct cytopathic activity, the virus induces a strong immune response mediated by both nuclear factor kappa light chain enhancer of activated B cells (NF-κB) and activation of nucleotide-binding oligomerization domain-like receptors (NLRs) [14]. The ensuing high level of proinflammatory cytokine production underlies the so-called cytokine storm, leading to severe symptoms and lesions such as vasculopathy, coagulopathy, and multiple organ injuries underlying mortality in most cases [15].

## Animal reservoirs and intermediate hosts of SARS-CoV-2

Since bats, and in particular horseshoe bats [16], are the main natural reservoirs of various coronaviruses (CoVs), from the very beginning it was hypothesized that they could also play the same role for SARS-CoV-2. Genomic sequencing and evolutionary analysis showed 96.2% sequence identity between SARS-CoV-2 and bat coronavirus (BatCoV) RaTG13 [4], suggesting that SARS-CoV-2 may have originated from bats [4, 17]. In particular, the bat species *Rhinolophus affinis* and *Rhinolophus malayanus* could be the original niche of SARS-CoV-2 [18]. However, SARS-CoV-2 contains mutations in its S glycoprotein and N protein sequences that differentiate it from BatCoV RaTG13. This suggests that the virus may have infected intermediate hosts, where it presumably mutated and acquired the ability to infect humans [19, 20]. This hypothesis is also supported by the fact that bats were not available for sale in the Huanan Seafood Market [12]. Few animals are under study to identify the putative intermediate host of the virus. Among them are pangolins, turtles, and snakes. Malaysian pangolins are nocturnal mammals found in South East Asia, but not in China, where they arrive via illegal smuggling, as they are highly sought after for traditional Chinese medicine and for their meat [18, 21]. Studies have revealed that a group of betacoronaviruses (β-CoVs) found in pangolins share only about 85-92% nucleotide sequence identity with SARS-CoV-2 [22–24]. Although this percentage of sequence identity is lower than that found between BatCoV RaTG13 and SARS-CoV-2, the pangolin CoV and SARS-CoV-2 have four of the five key amino acids of the RBD region in common, while bat RaTG13 CoV has only one of these amino acids in the RBD region [25]. Furthermore, the RBD of pangolin CoV is very similar to that of SARS-CoV-2 and shows a strong capacity to bind to human ACE2 [26]. These data, in addition to the observation that pangolins showed clinical signs, histological changes, and circulating antibodies, highlighted the possible role of pangolins in the interspecies jumping of SARS-CoV-2. Although pangolins are not indigenous to China but (as mentioned above) enter this country illegally, they probably share ecological niches with bats. Therefore, they may have been in contact with bats, thereby contracting SARS-CoV-2 infection as possible intermediate hosts [3, 26]. At the same time, phylogenetic analysis has ruled out the hypothesis that pangolins could be the natural hosts of SARS-CoV-2 [27].

Structural analysis of the binding of the SARS-CoV-2 RBD to the ACE2 receptor, together with evolutionary studies, has suggested that turtles (*Chrysemys picta bellii, Pelodiscus sinensis*, and *Chelonia mydas*) and snakes (*Bungarus multicinctus* and *Naja atra*) also could have served as intermediate hosts of SARS-CoV-2 [20, 28, 29]. However, Luan *et al.* reported that, in both snakes and turtles, ACE2 is unable to bind to the S protein of SARS-CoV-2, leading to the conclusion that these animals are unlikely to have served as intermediate hosts for the virus [30]. The identification of the natural and intermediate hosts that allowed inter-species jumping of the virus to humans is still an open issue.

## Occurrence of SARS-CoV-2 in animals

SARS-CoV-2 has spread rapidly across all continents, finding a receptive population in the human species, allowing efficient intraspecies transmission. With high levels of circulation among humans, the virus may occasionally be transmitted from humans to animals that share the same environment, highlighting the need for surveillance in a One Health context.

## Companion animals

Pets, such as dogs and cats, are often in close contact with humans. As a result of this close contact, the World Organization for Animal Health (OIE) has reported isolated cases of pets testing positive for COVID-19.

### Dogs

The first dogs testing positive for COVID-19 were identified in Hong Kong between February and March 2020. Twenty-seven dogs whose owners had contracted COVID-19 were tested, and only two (a 17-year-old Pomeranian and a 2.5-year-old German shepherd) tested positive for SARS-CoV-2 RNA in nasal and oral swabs [31–33]. After few days/weeks, neutralizing antibodies were detected in blood samples of the two dogs [31]. Neutralizing antibodies against SARS-CoV-2 were detected in other dogs belonging to COVID-19-positive owners in the Netherlands and in New York State (USA) [34]. A study was conducted by Patterson *et al.* between March and May 2020 on dogs from Italian families. Oropharyngeal, nasal, and/or rectal swabs were collected from 314 dogs, none of which tested positive for SARS-CoV-2 RNA. SARS-CoV-2-neutralizing antibodies were detected in 15 dogs (3.3%, 15/451), with titers ranging from 1:20 to 1:160. None of these animals displayed respiratory signs at the time of sampling [35]. A serological survey conducted by Colitti and colleagues found that some dogs in northern Italy, one of the most heavily affected areas in the world, tested positive for SARS-CoV-2. Moreover, that study showed an association between seropositivity and length of exposure to an infected owner, suggesting that the development of antibodies in pets could be a consequence of virus transmission from their owners [36]. In all cases, the infection in these animals was restricted to the upper respiratory tract, and they showed no apparent capability to transmit the virus to humans or other animals [37]. These observations were confirmed by experimental infection of dogs. In fact, studies conducted on experimentally infected dogs have shown that these animals do not shed the virus after infection but seroconvert and mount a neutralizing, antiviral antibody response [38, 39].

### Cats

Pet cats were also tested for antibodies against SARS-CoV-2 using ELISA, virus neutralization test (VNT), and Western blot. In particular, from January to March 2020 in the city of Wuhan, 15 out of 102 cats were positive by ELISA, and a further 11 were positive by VNT [40]. In addition to the antibody tests, the animals were also swabbed, but none of them tested positive [40]. In mid-March 2020 in Belgium and Hong Kong, SARS-CoV-2 RNA was detected by RT-qPCR in samples from two cats presenting with diarrhoea, vomiting, and labored breathing [41, 42]. Patterson *et al.* conducted a study in Italy on 180 cats, all of which tested negative for SARS-CoV-2 RNA, while SARS-CoV-2-neutralizing antibodies were detected in 11 cats (5.8%, 11/191), with titers ranging from 1:20 to 1:1280 [35]. A very low percentage (around 0.7%) of antibody-positive samples was observed in Germany in a study of 920 cats that were randomly tested [43]. On April 2020, two cats from New York State (USA), both presenting with sneezing and nasal discharge, tested positive for SARS-CoV-2 by RT-qPCR [44]. In Spain, only one female cat already suffering from other diseases (chronic feline gingiva-stomatitis, feline idiopathic cystitis, chronic kidney disease, and feline asthmatic bronchitis), out of eight belonging to COVID-19-diseased persons, was oropharyngeal swab positive but fecal swab negative [45]. In France, a study on a small cohort of veterinary students and their pets in close contact with COVID-19 patients revealed that three cats had respiratory and gastrointestinal signs, but none tested positive for viral RNA [46].

On 19 March 2021, the Istituto Zooprofilattico Sperimentale del Piemonte, Liguria e Valle d’Aosta (Italy) reported the presence of the English variant of SARS-CoV-2 (lineage B.1.1.7) in an 8-year-old male cat with respiratory symptoms, living in Novara (Piedmont) in a domestic setting, where the owners were in isolation [47].

Experimental infection has been reported in subadult (3- to 18-month-old), juvenile (1- to 3-month-old) [39], and adult cats (5- to 8-year-old) [38]. Similar to a natural infection, this generally results in mild respiratory symptoms, with young cats being more susceptible to SARS-CoV-2 [39]. The antibody response observed in cats could be the result of prior exposure to feline coronavirus (FCoV; genus *Alphacoronavirus*), which demands careful interpretation of serological testing, where a positive result could be due to cross-reactivity. Moreover, the possible cross-protection of FCoV-specific antibodies against SARS-CoV-2 infection is still debated. Finally, different studies have suggested the possible direct transmission of SARS-CoV-2 between cats [48, 49]. Cats could represent an important reservoir given their habit of wandering around different houses and in the wild, but studies have suggested that they remain infectious for a short time [38]. In addition, a study conducted by Gaudreault and colleagues demonstrated that cats develop a robust neutralizing antibody response that provides partial immune protection against reinfection [50].

### Ferrets

The results of experimental infections of ferrets via the intranasal route [39, 51] were similar to those observed in cats, characterized by evidence of upper respiratory airway infection with mild clinical signs, elimination of the virus with feces, and evidence of conspecific transmission of the virus [39, 51]. SARS-CoV-2 can replicate in the upper respiratory tract of ferrets, but replication in other organs has not been detected [39, 51]. The transmission of the virus in this species can occur both directly and indirectly, but the direct way leads to the development of further evident symptoms, such as increased temperature and decreased activity, as observed in humans [51–53]. In addition to being susceptible to experimental infection, ferrets have also been shown to be susceptible to natural infection. Indeed, a surveillance study conducted in Spain showed that 8.4% of ferrets kept as pets or working animals for rabbit hunting tested positive for SARS-CoV-2 viral RNA in nasal or rectal swabs [54]. Finally, a further study found that, of 127 domestic ferrets tested, two showed antibodies to SARS-CoV-2 [55].

## Livestock animals

### Poultry

Schlottau *et al.* inoculated chickens oculo- and oronasally to assess their susceptibility to SARS-CoV-2. No injected animal showed clinical signs, and all swabs and organ samples were negative for viral RNA. Also, none of the animals seroconverted [37, 51]. In another experiment, chickens, turkeys, ducks, quails, and geese were inoculated with SARS-CoV-2. None of the inoculated animals showed clinical signs, viral RNA was not detected in the swabs, and antibodies against SARS-CoV-2 were not detected in any of the tested animals [37, 52]. These studies suggest that poultry are not susceptible to SARS-CoV-2 infection and that the virus cannot be transmitted to humans, or vice versa from humans to poultry.

### Pigs

Two different experiments were conducted on pigs to test their susceptibility to SARS-CoV-2 infection. The results showed that neither viral RNA nor antibodies were detected in the animals, either inoculated or in contact with infected individuals. This indicated that swine are not susceptible to SARS-CoV-2 [39, 53]. In another study on piglets inoculated intranasally, intratracheally, intramuscularly, and intravenously, it was found that they did not develop infection following inoculation. However, animals inoculated intramuscularly or intravenously seroconverted 2-3 weeks after infection [56]. Yet, to our knowledge, there has been no control study in pigs to discriminate between antibody responses to live replicating virus and those to viral proteins of inactivated SARS-CoV-2. In this respect, Meekins and colleagues conducted an *in vitro* study where the ability of SARS-CoV-2 to infect porcine testis and porcine kidney cell lines (PK-15) was observed [57]. In contrast, the same authors, in an *in vivo* study, observed that none of nine pigs infected orally, intranasally, or intratracheally developed clinical signs, viral replication, or a specific antibody response at 4, 8, and 21 days postinfection (dpi) [57]. Recently, in a study involving 16 oro-nasally infected domestic pigs, clinical signs, including eye discharge, nasal discharge, and cough, were only detected during the first 3 dpi. Viral RNA was detected in nasal washes of two pigs at 3 dpi. Antibody titres in serum were found in only two animals at 11-15 dpi [58]. Recently, Sikkema and colleagues, in order to assess the risk of SARS-CoV-2 infection, transmission, and reservoir development in swine, combined results of one experimental and two observational studies, showing that although sporadic infections in the field cannot be excluded, large-scale transmission of SARS-CoV-2 among pigs is unlikely [59].

On the whole, the susceptibility of pigs to SARS-CoV-2 is highly contentious, and there is still no clue about the possible role of a previous exposure to porcine respiratory coronavirus (PRCV) and, most importantly, to porcine epidemic diarrhea virus (PEDV), which has been widespread in both the USA and Europe in recent years.

## Domestic ruminants

Only experimental infections have been documented in ruminants. In the first study, calves were infected intranasally with SARS-CoV-2 and did not show any clinical signs of disease [60]. Viral replication was evident in only two out of six calves, as confirmed by positive results in RT real-time PCR in nasal swabs only, whereas seroconversion was evident in a single animal [60]. The authors did not observe intraspecies transmission to other cattle housed in contact with the infected cattle [60]. The study also demonstrated that pre-existing infections with BoCoV did not protect the animals [60]. The capability of SARS-CoV-2 to infect bovine tissues was also assessed using *ex vivo* organ cultures, demonstrating that respiratory tissues of cattle and sheep allow the replication of the virus, unlike pig tissues [61]. More recently, another experiment was conducted by Falkenberg and colleagues on six colostrum-deprived calves approximately 6 weeks of age [62]. They were inoculated intratracheally or intravenously to assess viral shedding in nasal, urine, and rectal swab samples, whereas blood samples were collected to investigate viremia and seroconversion; tissue samples were also harvested during necropsy. SARS-CoV-2 RNA was only detected in two nasal swab samples collected on days 3 and 10 post-inoculation in two calves. The viral nucleic acid load in these samples was low, and infectious viral particles were not recovered from the samples. These results suggest that there was no productive replication of SARS-CoV-2 in calves after intratracheal and intravenous inoculation [62]. In any case, the data available so far call for a careful investigation into natural SARS-CoV-2 infection on ruminant farms and into a possible presence of the virus in slaughterhouses, where the risk of transmission to the personnel is of some concern.

## Minks and wild animals

Minks are associated with large-scale SARS-CoV-2 infection. Indeed, at the end of April 2020, on a Dutch farm with 13,000 minks, two of them tested positive for SARS-CoV-2. The infection rapidly spread throughout the farm, with a large number of animals clinically affected [63]. The transmission of the virus from an infected worker of the farm to the animals was suspected [63]. Minks showed clinical signs ranging from nasal exudate to severe respiratory syndrome, together with gastrointestinal disorders [64]. Several animals died, and the necropsies revealed severe pneumonia. Viral infection was also found on other mink farms in Italy [65], Denmark [66], Spain [67], Sweden [68], Greece, and United States [69]. Genetic and epidemiologic investigations demonstrated animal-to-human and human-to-animal transmission of the virus [70]. These data cause concerns about the possible infection of wild mustelids, which could become permanent reservoirs of the virus [71]. Indeed, in October 2020 a wild mink in Utah (USA) tested positive, resulting in the first case of infection in wild animals [72]. The possibility that a wild animal turns into a SARS-CoV-2 reservoir raises more concern than the same case in a domesticated animal, which can be easily checked through quarantine, vaccination, or culling [73]. Very few data are available on the real resistance or susceptibility of wild animal species, suggesting that proper sanitary precautions should be adopted by humans when interacting with wild mammals [74]. In this respect, it would be interesting to test the susceptibility of bat species endemic on continents other than Asia. Two experimental infection studies on fruit bats (*Rousettus aegyptiacus*) and big brown bats (*Eptesicus fuscus*), respectively, yielded opposite results. Fruit bats were transiently infected with no clinical signs [75], whereas big brown bats did not show any signs of infection and seemed resistant to SARS-CoV-2 [76].

## Captive animals

In early April 2020, at the Bronx Zoo in New York (USA), animals including Malayan tigers, Siberian tigers, and African lions showed respiratory signs, and the United States Department of Agriculture (USDA) reported that a swab sample from a 4-year-old Malaysian tiger tested positive for SARS-CoV-2 by RT-qPCR [77]. An African lion was also confirmed to be positive [78]. After a few days, stool samples from the animals that showed clinical signs tested positive for SARS-CoV-2 by RT-qPCR [79]. The hypothesis was put forward that an asymptomatic employee of the zoo might have infected the Malayan tiger, resulting in the first reported case of a non-domestic animal being infected by a human [79].

In addition to the animal found positive in the Bronx Zoo in New York (USA), further reports of virus-positive animals are available: three tigers at the Knoxville Zoo in Tennessee (USA) [80], three snow leopards at the Jefferson Zoo in Kentucky (USA) [80], and four lions at the Barcelona Zoo in Spain [81]. All of these animals showed respiratory signs. Moreover, a cougar at Johannesburg Zoo in South Africa tested positive but did not show any clinical signs [82].

Nasal and oral swabs from experimentally infected raccoon dogs contained viral genomic RNA, and these animals transmitted the virus to contact animals, suggesting their role as potential reservoirs [83].

In January 2021, some captive gorillas at the San Diego Zoo (USA) showed respiratory signs, and SARS-CoV-2 RNA was found in their feces. Also in this case, an asymptomatic member of the wildlife team was suspected to be responsible for the infection of the apes [84]. All of the animals showed mild signs of disease; this event may cause concerns for the wild endangered great apes, which cannot be cared for as they can in captivity.

A recent study demonstrated the susceptibility of white-tailed deer (*Odocoileus virginianus*) to SARS-CoV-2 infection. The experimentally infected animals developed subclinical infection and eliminated viral particles in nasal secretions, thereby transmitting the virus to contact animals. Viral genomic RNA was detected in different organs, and neutralizing antibodies were present in all the experimentally infected and contact deer [85].

## Laboratory animals

The spread of the COVID-19 pandemic demands a model that could faithfully reproduce the biological cycle of the virus and the pathogenesis of the disease in humans. Cell lines and organoids have been used for this purpose, but because of the complex pathophysiology of SARS-CoV-2 infection, animal models have to be used as well [86]. For this purpose, several animal species have been included in these studies.

### Mice

Wild-type laboratory mice do not show susceptibility to SARS-CoV-2 infection, which is presumably due to significant differences between the murine and human ACE2 molecules [4]. To overcome this problem, humanized mice expressing human ACE2 (hACE2) were generated [87, 88]. Experiments in humanized mice expressing hACE2 showed that, following infection with SARS-CoV-2, high levels of viral replication were detected in the lungs, with spread to other organs [89, 90]. Humanized mice expressing hACE2 were generated by different approaches (transgenic mice, use of adenovirus and human cytokeratin 18-based vectors), and in the last two years, more than 150 articles have described their use in studies on pathogenesis, infection, immune response, therapies, and vaccines against SARS-CoV-2. Several studies have shown that hACE2 transgenic mice infected with SARS-CoV-2 can successfully mimic human COVID-19 [91–93]. A study using clustered regularly interspaced short palindromic repeats by repetitive/Cas9 knock-in technology to generate humanized mouse strains expressing hACE2 revealed that hACE2 mice supported SARS-CoV-2 replication in lung Clara cells and macrophages and showed symptoms that were similar to those of COVID-19 patients [89]. An experiment conducted by Dinnon and colleagues showed that the severity of the disease is related to the age of the mouse. By infecting SARS-CoV-2-adapted young, adult, and elderly BALB/c mice, respectively, they showed that virus replication occurred in both the upper and lower airways, with more-severe disease in the older mice [91]. Moreover, SARS-CoV-2 infection in BALB/c mice encoding hACE2 caused lung pathology, weight loss, and viral pneumonia, and high levels of viral RNA were detected in the lungs [94].

### Hamsters

Hamsters have been used successfully to assess SARS-CoV replication [95, 96]. Accordingly, they were deemed to be a good model for SARS-CoV-2 infection as well. The experiments conducted so far have shown that, following virus inoculation, clinical signs such as lethargy, ruffled fur, and weight loss occurred in Syrian Golden hamsters, with subsequent development of the disease and detection of viral RNA [97–99]. Furthermore, clinical features, virus replication kinetics, histopathological changes, and immune responses in SARS-CoV-2-infected Syrian hamsters were similar to those described in human patients affected by COVID-19 [97, 98, 100]. Intraspecific transmission has also been demonstrated [98]. In addition, Lee and colleagues reported that oral inoculation, compared to intranasal inoculation of SARS-CoV-2 in Syrian hamsters, produced milder symptoms and histological lesions, as well as reduced viral shedding [101]. One study also showed that the severity of SARS-CoV-2 infection in Syrian hamsters correlated with the age of the animals, with older hamsters showing more-pronounced weight loss, more-severe histological lung lesions, and delayed recovery at 14 dpi compared to younger animals [102]. As hamsters proved to be a good small-animal model for studying the virus, the roles of types I and III IFNs in the pathogenesis of SARS-CoV-2 infection were investigated. Experiments performed on wild-type, STAT2-/- (lacking type I and III IFN signaling), and IL28R-α -/- (lacking IFN type III signaling) hamsters showed that STAT2 signaling is a double-edged sword: on the one hand, it restricts viral dissemination, but, on the other hand, it causes severe pneumonia in SARS-CoV-2-infected hamsters [103].

## Non-human primates

Several non-human primates have been experimentally infected in order to establish a suitable non-human primate model of COVID-19. In particular, Old World monkeys (*Macaca mulatta* and *Macaca fascicularis*) and New World monkeys (*Callithrix jacchus*) were tested, all of which developed signs such as fever and weight loss, without respiratory symptoms. Viral RNA was detected in swab and blood samples from all animals. *M. mulatta* was the species most susceptible to SARS-CoV-2 infection in terms of inflammatory cytokine expression and formation of pathological lesions in the lung, representing the most suitable model of COVID-19 [104]. Other studies were then conducted on *M. mulatta*. These animals were infected through the intratracheal route [105], the intranasal route [106], the ocular route [107], the intragastric route [108], or a combination of the intratracheal, intranasal, ocular, and oral routes [109]. The results obtained were similar to those of Lu and colleagues, demonstrating that this non-human primate model can be considered for reproducing COVID-19 of moderate severity [107]. African green monkeys (*Chlorocebus Sabaeus*) were also used as a model of SARS-CoV-2 infection [110, 111]. These animals developed fever, loss of appetite, and respiratory signs. Moreover, viral RNA and infectious virus were detected in nasal swabs, and ultimately, all of the animals seroconverted and exhibited a specific cell-mediated immune response. The results obtained in these studies suggest that the African green monkey model reflects the pathology of severe human COVID-19 cases more accurately than other non-human primate models [110, 111].

**Fig. 1:**
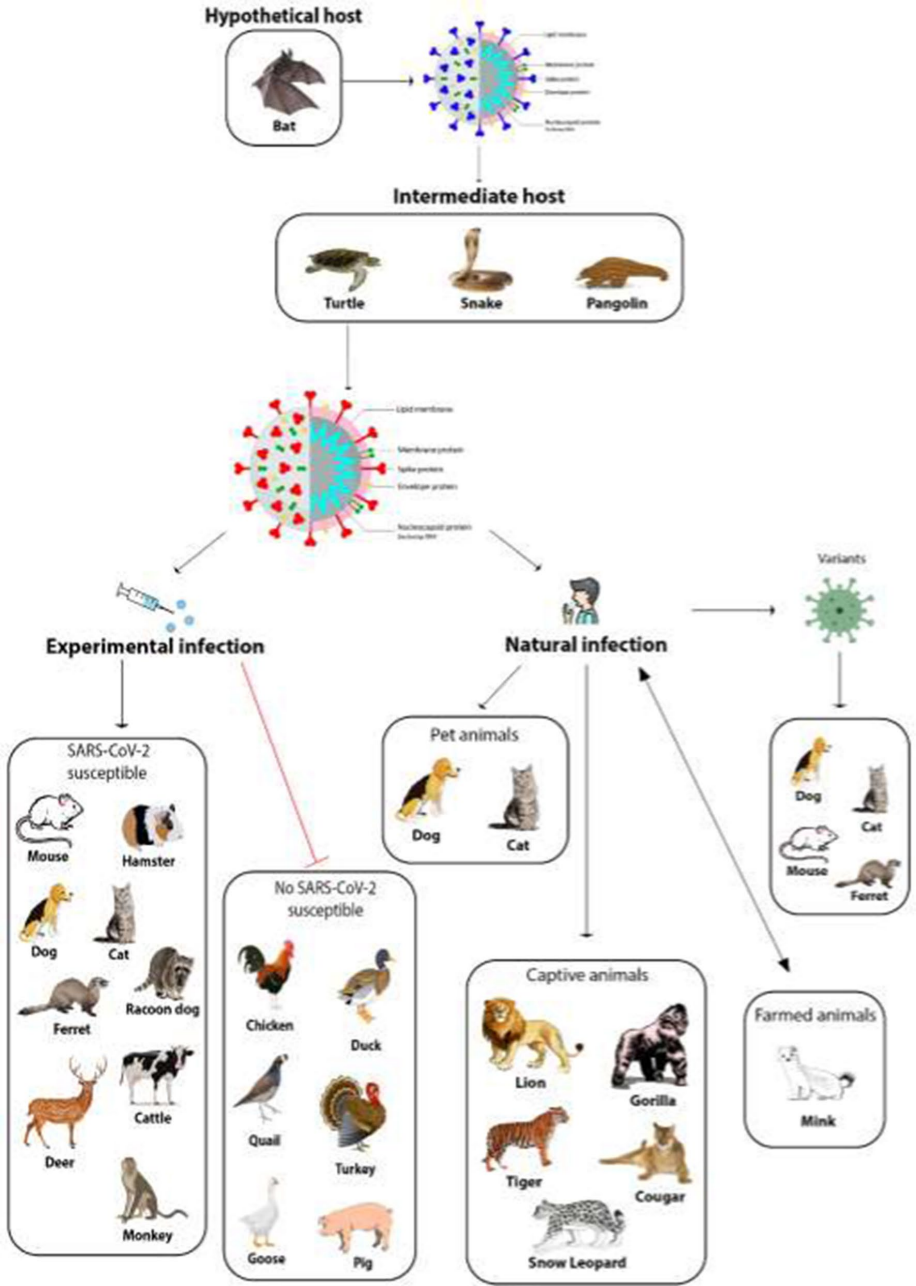
SARS-CoV-2 transmission among different animals. The most widely accepted hypothesis is that SARS-CoV-2 was derived from a bat coronavirus after a modification in a putative intermediate host, where it acquired the capability to infect humans. The wide circulation of the virus among humans caused a pandemic, and it is plausible that infected humans may have transmitted the virus to different animal species. In order to better understand the role of animals in the epidemiology of SARS-CoV-2 and to establish appropriate animal models, several species have been experimentally infected, but not all of them were found to be permissive for the infection. To date, only minks seem to be able to transmit SARS-CoV-2 infection to humans. Greater attention should be devoted to monitoring new variants of SARS-CoV-2 because of their potential to acquire the ability to infect domestic or wild animals, which could potentially serve as reservoirs for the virus.

Figure 1 summarizes the possible circulation of SARS-CoV-2 among different animals based on available data from natural and experimental infections.

Table 1 summarizes the degree of susceptibility to SARS-CoV-2 infection of different animals and their capability to transmit it, based on available data from natural and experimental infections.

**Table 1:** Susceptibility to SARS-CoV-2 infection and transmissibility in different animals under natural and/or experimental conditions

Animals	Type of infection	Susceptibility to infection	Transmission
Companion animals			
Dogs	Natural and experimental	Low	No
Cats	Natural and experimental	High	Between cats
Ferrets	Natural and experimental	High	Between ferrets
Livestock animals			
Poultry (chicken, ducks, turkeys, quail and goose)	Experimental	None	No
Pigs	Experimental	Extremely low	No
Cattle	Experimental	Extremely low	No
Minks	Natural and experimental	High	Yes, between minks and from minks to humans
Captive animals			
Large cats (tigers, lions, snow leopards and pumas)	Natural	High	Yes, between animals
Gorillas	Natural	High	Yes
Raccoon dogs	Experimental	High	Yes, between raccoon dogs
White-tailed deer	Experimental	High	Yes, to other white-tailed deer
Laboratory animals			
Humanized mice expressing hACE2	Experimental	High	Yes, between humanized mice expressing hACE2
Syrian golden hamsters	Experimental	High	Yes, between hamsters
Non-human primates (*Macaca mulatta, Macaca fascicularis, Callithrix jacchus, Chlorocebus Sabaeus)*	Experimental	High	Yes

## COVID-19 vaccines for animals

As part of a One Health approach, the threat of animal-to-human transmission and the relevant rise of mutant virus variants should be adequately dealt with. In this respect, Russian researchers of the Federal Service for Veterinary and Phytosanitary Surveillance have developed the first COVID-19 vaccine for animals (*WION*, n.d.) [112]. The vaccine, known as Carnivac-Cov, was designed for carnivores and developed based on an inactivated vaccine platform (*Russia Beyond*, n.d.) [113]. Studies on the efficacy of this vaccine were initially conducted on ferrets, and then, after promising results, experimental trials were carried out on arctic foxes, cats, rats, minks, and other animals that are more susceptible to SARS-CoV-2 than ferrets. The data obtained so far suggest that Carnivac-Cov is safe and capable of inducing immunity in all of the animals tested in the study (*WION*, n.d.) [112]. The purpose of this veterinary vaccine against SARS-CoV-2 is to protect animals from infection and prevent the development of dangerous viral mutations.

## The contribution of animals to immunoprophylaxis of COVID-19

Different animal models that mimic the development of the disease in humans have been used to develop and evaluate vaccines, immunotherapy, and other possible therapies to combat SARS-CoV-2 infection. The rapid development of vaccines is made possible by the availability of appropriate laboratory animal models. Vaccines have proved especially useful in protecting against the development of lethal COVID-19 [114]. However, we are still in need of further preventive measures to complement the present vaccines and control the spread of SARS-CoV-2. Given the similarity of highly conserved structures in bovine coronavirus (BCoV) and SARS-CoV-2, and since neutralizing antibodies (NAbs) are able to block the entry of a pathogen into the cell and thus prevent infection [115, 116], finding efficient bovine NAbs that are able to block the entry of SARS-CoV-2 could be a promising prophylactic and/or therapeutic approach to fight against the pandemic. In this direction, some studies have been carried out recently. Arenas *et al.* have suggested that recognition of certain highly conserved motifs of viral proteins, in particular M and S2, by anti-BCoV antibodies present in milk would cause total or partial inactivation of SARS-CoV-2 [117]. Kangro and colleagues showed that the combination of antibodies derived from colostrum of cows immunized with the SARS-CoV-2 spike protein in an intranasal formulation can provide an efficient blockade against infection with SARS-CoV-2, including several of the known variants [118]. The use of bovine NAbs has previously been shown to be a potential strategy to combat HIV infections. A subset of some rare, broadly neutralizing antibodies (BNAbs) isolated from infected individuals has been shown to exhibit a long immunoglobulin heavy chain complementarity determining region 3 (CDR H3) [119]. This feature generates unique configurations of the antigen binding site that can thus engage conserved, but otherwise inaccessible, epitopes, thereby neutralizing many viral variants. Ultra-long CDR H3s are a common feature of the cow antibody repertoire. They are encoded by a single variable diversity recombination (VDJ regions) that is extensively diverse prior to antigen exposure [120, 121]. Given this evidence, a number of studies have been conducted on the efficacy of BNAbs against HIV. Among these, Sok and colleagues demonstrated that immunization of cows may provide a way to rapidly generate antibodies to pathogenic agents that have escaped human antibody responses [122]. Given that monoclonal antibodies cannot yet be produced easily and economically, other approaches are badly needed. In this context, different groups have undertaken studies to develop neutralizing nanobodies against the RDB of SARS-CoV-2 [123–126]. Nanobodies consist of variable domains of camelid (llama, alpaca, camel) heavy-chain-only antibodies (VHHs). They can be produced in prokaryotic systems with high yield and low production costs, they are easily modified, and are characterized by peculiar biological properties such as high tissue penetration capability and thermostability [127]. In particular, due to the possibility of easy modifications, nanobodies are very promising for the treatment of infections caused by new variants of SARS-CoV-2 [128]. Moreover, due to the urgency to develop new therapies against SARS-CoV-2, Salinas and colleagues investigated the preclinical safety and biodistribution of CoviFab (INM005), which is an RBD-specific F(ab′)2 fragment derived from equine polyclonal antibodies [129]. The results show that CoviFab is safe, since no adverse effects were observed in mice, and that it localizes and remains in the organs targeted by SARS-CoV-2 [129].

Although small-animal models are an important starting point for vaccine development and study, they often show variable success. In contrast, larger-animal models, such as pigs and non-human primates, can more accurately predict the efficacy of vaccines in humans [130, 131]. A study conducted by Graham and colleagues used both mice and pigs to evaluate the immunogenicity of either one or two doses of a COVID-19 vaccine candidate, ChAdOx1 nCoV-19 [132]. Data obtained in mice showed an immunogenicity profile at the upper end of the dose-response curve; this vaccination schedule may have saturated the immune response, and consequently, it did not allow the detection of crucial differences between prime-only and prime-boost regimens. On the other hand, the data obtained in pigs have shown that this animal model is useful for studying COVID-19 vaccines. Most importantly, T-cell responses were higher at day 42 in pigs that received a prime-boost vaccination compared to those on the prime-only schedule, and comparison of responses 14 days after the last immunization showed that the prime-boost treatment tended to induce a stronger response. In addition to that, SARS-CoV-2 neutralizing antibody titers in pigs after a single immunization appeared similar to those found in sera of humans following asymptomatic infection, while titers in pigs in the prime-boost group were similar to those found in sera of recovered COVID-19 patients [132].

## Conclusion

Given the high transmissibility and the zoonotic nature of COVID-19, it is necessary to investigate the role animals might play in SARS-CoV-2 epidemiology. Different studies have been performed to investigate the potential susceptibility of animals to infection, based on the similarity/homology of orthologous ACE2 proteins [20, 26, 28–30, 133, 134]. Indeed, the species (apes, felines, hamster, ferrets) whose ACE2 is most similar to the human one proved more susceptible *in vivo* to both natural and experimental infections [20, 26–28, 30, 133, 134].

Studies on companion animals seem to indicate cats as more susceptible than dogs to viral infection. They are mostly asymptomatic or paucisymptomatic and can transmit the virus to their conspecifics, but so far, there is no evidence of direct animal-to-human transmission.

All of the natural infections of animals reported so far (cats, dogs, tigers, gorillas, minks) probably occurred following contact with an asymptomatic person or with a virus-positive owner. Therefore, it is important to protect the pets of COVID-19 patients by limiting their exposure to their owners and possibly creating clear guidelines on the management of pets whose owners are affected by COVID-19.

The demonstration of the susceptibility of animals living in close contact with humans indicates the need for a One Health approach to the study and management of the pandemic. This means that more investigation is needed to elucidate the role of domestic and wild animals in the circulation of SARS-CoV-2. In particular, it is critical to understand the susceptibility of animals to SARS-CoV-2 in order to check the spread of the virus. Although no study has conclusively demonstrated that animals can transmit the virus to humans, there is increasing concern that animals, once infected, might pose a threat to humans. The experience on mink farms is a clear example of this risk. In addition, a new variant of the virus could find a wild animal species as permanent reservoir, keeping the virus circulating in the world or recombining with other coronaviruses present in the host. Moreover, in the One Health approach, the long experience of veterinary practitioners with animal coronavirus infections could not only support investigations on the origin and spread of SARS-CoV-2 but also guide future studies for the establishment of effective therapeutic protocols and the development of new efficacious vaccines for humans [134]. Finally, the demonstrated benefits of animal models for the development of active and passive immunization against SARS-CoV-2 make a case for large-scale concerted efforts at the international level.

Investigation of the SARS-CoV-2 pandemic has taught us that the virus spilled over from animals into humans and, through global movement of people and their contacts with domestic and peridomestic animals, it spread all over the word into a wide range of animal species. Interspecies transmission of the virus promotes its evolution and the appearance of new variants, as demonstrated by the mink model. Accordingly, the possible role of animals in the emergence of new virus variants needs to be carefully monitored [135, 136]. For example, Gu and colleagues demonstrated the adaptation of a variant of SARS-CoV-2 to BALB/c mice [137].

Interestingly, careful monitoring of the spread of SARS-CoV-2 among animals (domestic, captive and wild) has been initiated all over the world, and a surveillance program has been unified under the auspices of OIE, WHO, and the US Center for Disease Control and Prevention with the publication of a guide to surveying animals and monthly meetings with researchers in the field [73].

Finally, a note of caution should be put forward about misleading mass information. Although the origin of SARS-CoV-2 is still uncertain, mass media has disseminated the mistaken idea that bats are dangerous virus transmitters [138]. Public opinion about bats, influenced by incorrect interpretation of scientific data by mass and social media, could negatively impact the conservation of these animals. Bats play important roles, both in natural and human-modified ecosystems, such as reduction of insect spread, pollination, and dispersal of vegetal seeds; importantly, various bat species are endangered and need to be protected [138, 139].
